# Virtual and Interprofessional Objective Structured Clinical Examination in Dentistry and Dental Technology: Development and User Evaluations

**DOI:** 10.2196/44653

**Published:** 2024-01-17

**Authors:** MengWei Pang, YanLing Dong, XiaoHan Zhao, JiaWu Wan, Li Jiang, JinLin Song, Ping Ji, Lin Jiang

**Affiliations:** 1 Stomatological Hospital of Chongqing Medical University Chongqing China; 2 Chongqing Key Laboratory of Oral Diseases and Biomedical Sciences Chongqing China; 3 Chongqing Municipal Key Laboratory of Oral Biomedical Engineering of Higher Education Chongqing China; 4 College of Stomatology, Chongqing Medical University Chongqing China; 5 State Key Laboratory of Virtual Reality Technology and Systems, Beihang University Beijing China; 6 Beijing Unidraw Virtual Reality Technology Research Institute Co Ltd Beijing China

**Keywords:** dentist, dental technician, objective structured clinical examination, OSCE, interprofessional education, interprofessional collaborative practice

## Abstract

**Background:**

Interprofessional education (IPE) facilitates interprofessional collaborative practice (IPCP) to encourage teamwork among dental care professionals and is increasingly becoming a part of training programs for dental and dental technology students. However, the focus of previous IPE and IPCP studies has largely been on subjective student and instructor perceptions without including objective assessments of collaborative practice as an outcome measure.

**Objective:**

The purposes of this study were to develop the framework for a novel virtual and interprofessional objective structured clinical examination (viOSCE) applicable to dental and dental technology students, to assess the effectiveness of the framework as a tool for measuring the outcomes of IPE, and to promote IPCP among dental and dental technology students.

**Methods:**

The framework of the proposed novel viOSCE was developed using the modified Delphi method and then piloted. The lead researcher and a group of experts determined the content and scoring system. Subjective data were collected using the Readiness for Interprofessional Learning Scale and a self-made scale, and objective data were collected using examiner ratings. Data were analyzed using nonparametric tests.

**Results:**

We successfully developed a viOSCE framework applicable to dental and dental technology students. Of 50 students, 32 (64%) participated in the pilot study and completed the questionnaires. On the basis of the Readiness for Interprofessional Learning Scale, the subjective evaluation indicated that teamwork skills were improved, and the only statistically significant difference in participant motivation between the 2 professional groups was in the mutual evaluation scale (*P*=*.*004). For the viOSCE evaluation scale, the difference between the professional groups in removable prosthodontics was statistically significant, and a trend for negative correlation between subjective and objective scores was noted, but it was not statistically significant.

**Conclusions:**

The results confirm that viOSCE can be used as an objective evaluation tool to assess the outcomes of IPE and IPCP. This study also revealed an interesting relationship between mutual evaluation and IPCP results, further demonstrating that the IPE and IPCP results urgently need to be supplemented with objective evaluation tools. Therefore, the implementation of viOSCE as part of a large and more complete objective structured clinical examination to test the ability of students to meet undergraduate graduation requirements will be the focus of our future studies.

## Introduction

### Interprofessional Collaboration Between Dentists and Dental Technicians

Conflicts are part of the life of any organization, and the dental professions are not spared. Jurisdictional battles and supremacy struggles are not alien to dentistry [1]. Unfortunately, despite the obvious reported benefits of interprofessional education (IPE) for interprofessional collaborative practice (IPCP) [2], there is a paucity of data about IPE to promote IPCP among dental professionals. Dentists and dental technicians need to communicate effectively and contribute their professional skills to ensure that they make decisions that are in the best interests of their patients [3]. A clear understanding of the interactions of the dental care team can promote teamwork [4], establish cooperative goals [5], encourage mutual respect [6], and promote IPCP between dental students and dental technology students [7].

IPE encourages teamwork among dental care professionals [8-11] and is increasingly becoming a part of the training programs for dental and dental technology students [12-17]; however, gaps still remain. Perhaps, the largest gaps are owing to the predominant focus of previous studies regarding IPE and IPCP on student and instructor perceptions and a lack of objective assessment of collaborative practice as an outcome measure [18,19]. This marked gap has necessitated the development of a conceptual framework to evaluate the impact of IPE on IPCP to strengthen the evidence for IPE as a tool to improve IPCP between dental and dental technology students [20].

### The Objective Structured Clinical Examination

The objective structured clinical examination (OSCE) is an assessment tool based on the principles of objectivity and standardization, in which individual students move through a series of time-limited stations in a circuit for the purpose of assessment of professional performance in a simulated environment. At each station, the student is assessed and marked against standardized scoring rubrics by trained assessors [21]. OSCE has been widely adopted as a summative assessment in the medical undergraduate curriculum and is universally accepted as the gold standard for assessing clinical competence in dental education [22,23]; furthermore, its effectiveness has been confirmed by several studies [24-26]. On the basis of the extensive application of OSCE, the interprofessional OSCE (iOSCE) was initially developed to simulate IPCP [27]. Unlike conventional OSCE, iOSCE involves students from different professions, encourages students to work as a team, and requires the entire team to participate in all tasks [28]. This is performed to objectively evaluate the results of IPE. Within this framework, several variations of iOSCE have been developed to accommodate the training needs of health care teams built to address different disease categories (team OSCE [29,30], group OSCE [31,32], interprofessional team OSCE [28,33], etc). These iOSCE variants can be roughly divided into synchronous [33-36] and asynchronous [29,37] task-based variants. A team working in an operating room typically works synchronously, whereas health care teams of dentists and dental technicians typically work asynchronously. Although the use of iOSCE in medical education has been extensively reported [27-30,38-42], to the best of our knowledge, the use of iOSCE for asynchronous work, especially within dentistry and dental technology cross-professional education, has not been reported.

Although iOSCE may provide an ideal solution for dental and dental technology students to perform IPCP simulation based on real patient cases, the COVID-19 pandemic [43] highlighted the limitations of this traditional approach. For example, a plaster model generated from a clinical case and passed multiple times among students and examiners may pose a risk of infection. In addition, diagnostic stations are usually set up to facilitate OSCE. A station is typically equipped with a trained, standardized patient, and the students complete the diagnosis by asking questions and examining this standardized patient. The risk of infection at this type of station was heightened during the pandemic. Nevertheless, compared with the traditional OSCE, iOSCEs are more time consuming and resource intensive [44,45], especially in dental education; hence, a virtual approach, as developed and piloted in this study, is justified [46,47]. Notably, the conventional virtual OSCE (vOSCE) has been described as a method of performing OSCE using internet technology in medicine [47-49]. The major reason for this technological approach was the scattered nature of the locations of students requiring assessment. However, this approach does not fully leverage virtual technology in dentistry. The integration of digital dental technologies and cloud-based dental laboratory workflows could be practiced within the vOSCE framework [50], which now also forms a professional core course in dental technology education [51-53]. The development of iOSCE based on virtual technology could facilitate the inclusion of digital dental technology in the blueprint design of examination stations. This combination could simulate the critical needs of present-day dental laboratories and promote students’ improved perception about the current demands of the profession.

### Objective

To address these research gaps, this study presented a new virtual iOSCE (viOSCE) to objectively assess the effectiveness of IPE as a tool to promote IPCP among dental and dental technology students. We have described the development and piloting of a viOSCE framework and its virtual techniques to validate the user-friendliness of IPE and document its effect on IPCP among dental and dental technology students. Data from both subjective and objective evaluations were collected, and their correlation was assessed.

## Methods

### Development of viOSCE

The principal investigator (PI) first limited the viOSCE knowledge to content related to the prosthodontics course. Content related to implantology and orthodontics was excluded because it is not part of the core undergraduate coursework for dental or dental technology students. On the basis of the Association for Medical Education in Europe guide [54], a modified Delphi method was used to generate content for viOSCE. The Delphi method is a decision-making process that uses expert opinion, gathered in the form of a survey, under the guidance and direction of the PI to reach group consensus through collaboration, independent analysis, and iteration [55]; this process is the most frequently used method to generate content for OSCEs [54]. The panel of experts in this study consisted of 9 instructors (including the PI) from the College of Stomatology, Chongqing Medical University. All 9 instructors had prosthodontics teaching experience and digital technology practical teaching experience with undergraduate dental and dental technology students. They had also participated in the design and examiner training for traditional OSCE, but only the PI had experience in IPE and vOSCE design.

**Figure 1:**
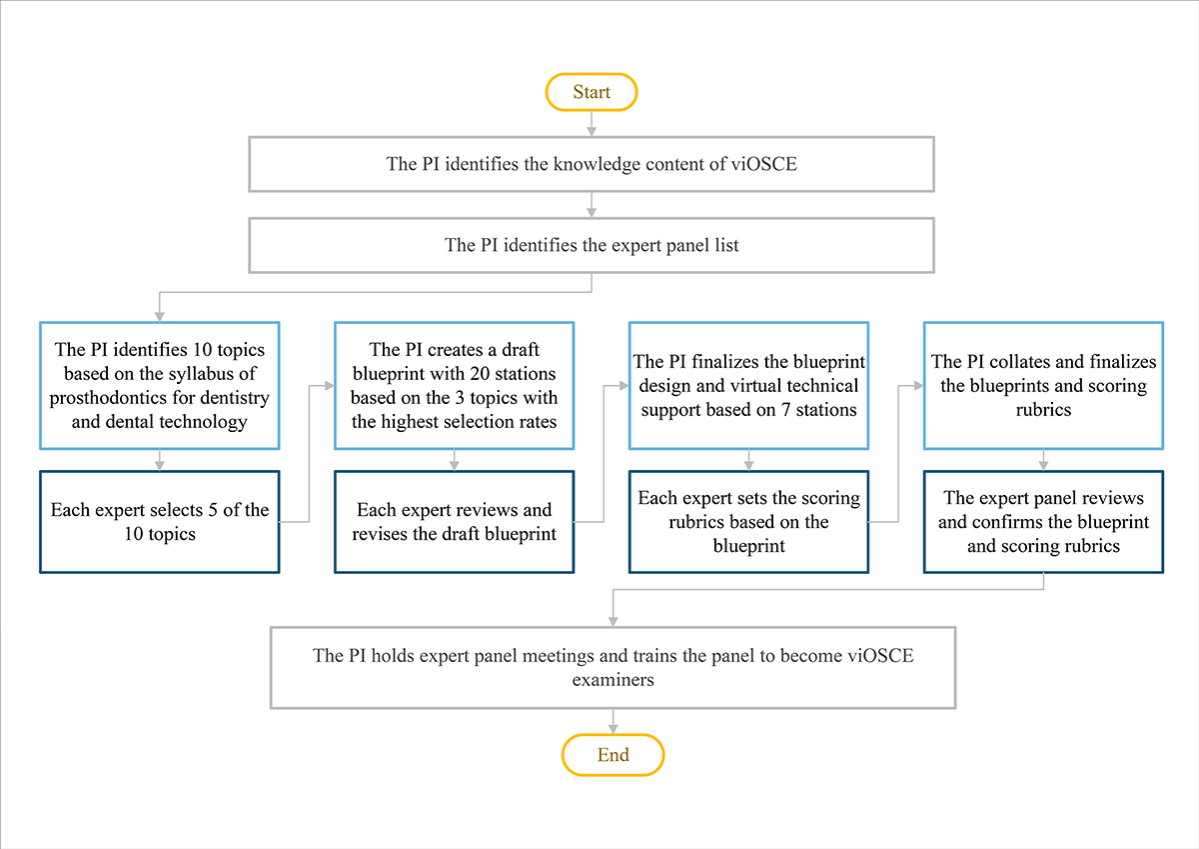
The viOSCE development process based on the modified Delphi method. PI: principal investigator; viOSCE: virtual and interprofessional objective structured clinical examination.

In this study, there were 4 iterations (rounds) before the viOSCE station design was finalized. In the first round, the PI identified 10 potential topics for viOSCE based on the syllabus of the prosthodontics course for dentistry and dental technology students, gave initial suggestions for the station design, and created a manuscript that was emailed to the panel of experts. Each expert independently gave their opinion and selected 5 topics that they considered as the most important in the syllabus and the most suitable for assessment using viOSCE. In the second round, the PI identified 3 topics with the highest selection rate based on the expert feedback and designed draft blueprints for 20 stations based on the top 3 selected topics using existing virtual technology support. These were sent to the expert panel via email. The expert panel commented about the potential effectiveness of interprofessional collaboration at the stations, made necessary corrections, and returned the design drafts to the PI. In the third round, the PI summarized all the changes made by the expert panel and, finally, decided on 7 stations based on the availability of virtual technology and the time to be spent on the stations within the allotted time frame of the examination. Stations consuming a lot of time, requiring multiple devices for support, or requiring very large spaces were rejected. Next, the selected viOSCE station blueprint design was completed, the virtual technical support was finalized, and the PI sent the final viOSCE station blueprint to the expert panel via email. The expert panel created the scoring rubrics based on the final viOSCE station blueprint, and these were returned to the PI for finalization. In the final round, the PI compiled all the information and met with the group to get a consensus regarding the viOSCE station blueprint and scoring rubrics. Once all the experts approved the viOSCE test station blueprint and scoring rubrics, the PI declared the viOSCE design as complete and declared the panel of experts the viOSCE examiner panel (Figure 1).

### The viOSCE Framework

**Figure 2:**
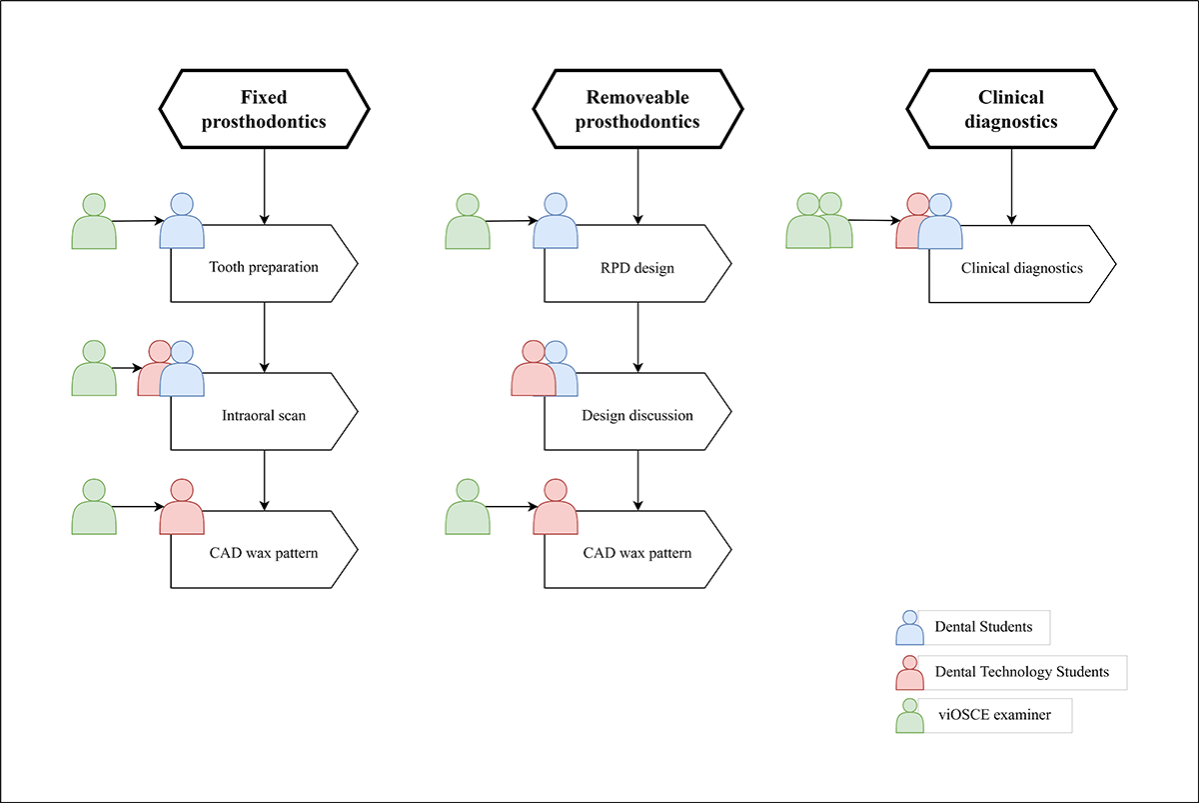
The framework of the viOSCE. CAD: computer-aided design; RPD: removable partial denture; viOSCE: virtual and interprofessional objective structured clinical examination.

The developed viOSCE framework consisted of 3 topics, namely, fixed prosthodontics, removable prosthodontics, and clinical diagnostics. There were 7 collaborative examination stations consisting of 4 asynchronous and 3 synchronous stations. All these stations were designed and developed using the Delphi method (Figure 2).

**Figure 3:**
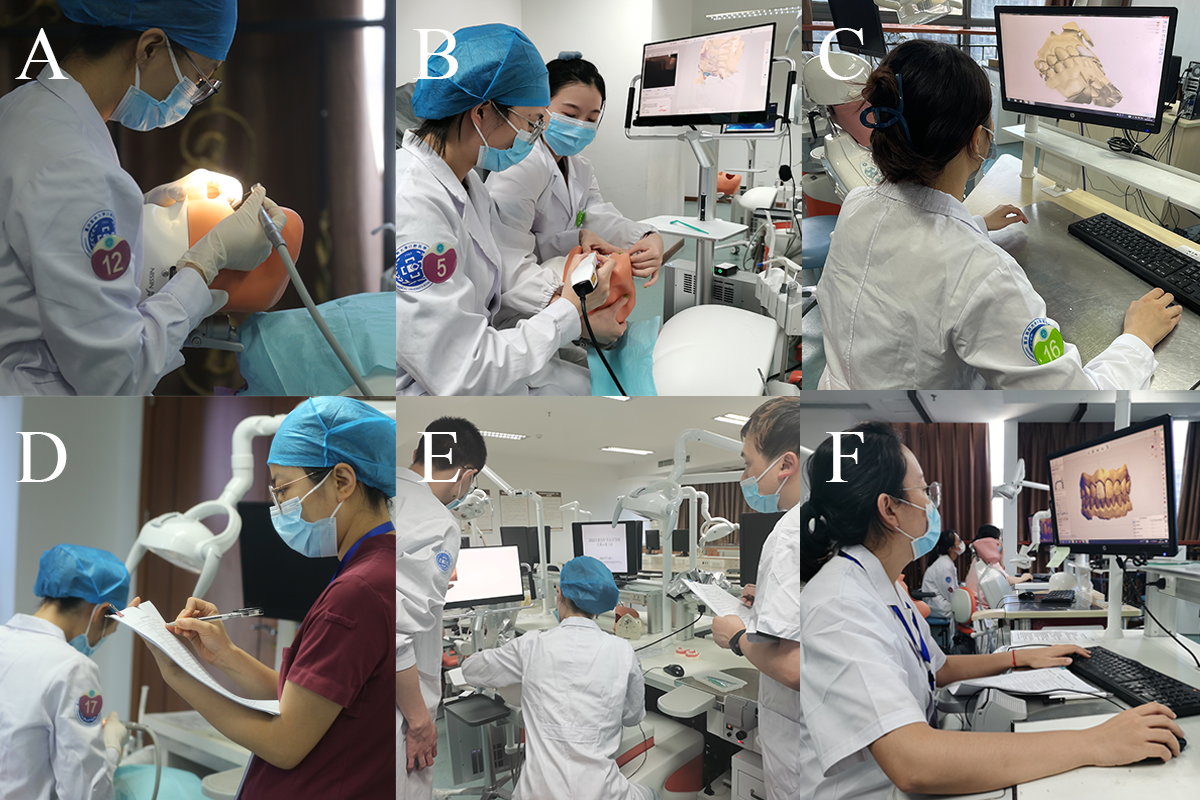
The fixed prosthodontics stations of the viOSCE. (A) A dental student prepared tooth 8 on the simulator. (B) Dental and dental technology students scanned the preparation using an intraoral scanner. (C) A dental technology student created a digital wax pattern using the computer-aided design system. (D) A viOSCE examiner scored the preparation process and results. (E) A viOSCE examiner scored the intraoral scanning process and results. (F) A viOSCE examiner scored the digital wax patterns. viOSCE: virtual and interprofessional objective structured clinical examination.

At the fixed prosthodontic stations, the dental student prepared tooth 8 (maxillary right central incisor) on the simulator (Nissan Dental Products) and then worked with the dental technology student to scan the preparations using an intraoral scanner (Panda P2; Freqty Technology). The dental and dental technology students at the intraoral scanning station worked collaboratively. The dental student performed an intraoral scan task, and the dental technology student observed the scan results to determine whether they could be used for the computer-aided design (CAD) wax pattern station. After obtaining a digital model, the dental technology student used a CAD system (Dental system; 3shape) to design a single crown on the digital model of the preparation. Individual scoring rubrics were designed for tooth preparation, intraoral scan, and CAD wax pattern. The 3 examiners scored each of the 3 stations (Figure 3 and Tables 1-3).

**Table 1:** The scoring rubric used to assess the tooth preparation stations.

Scoring component	Points	Details
Preparation before operation	5	Infection control was correctly performed Correct adjustment of phantom head position and lighting The operating position is correct
Fine motor skills	15	Holds the handpiece correctly Fulcrum stability Correct use of the mouth mirror to reflect areas to be operated under indirect vision Accurate application of burs
Preparation during operation	15	Operation sequence correctly performed Placement of depth orientation grooves Labial surface prepared in 2 planes
Incisal reduction	10	1.5-2 mm Formed a small bevel inclined 45° to the lingual side
Axial reduction	15	2 mm for the labial surface 1 mm for the proximal surface 0.7-1 mm for the lingual surface
2-plane reduction	5	Labial surface forms 2 planes and has rounded line angles and point angles
Taper	5	Retentive walls: 6°-10°
Margin placement	10	Margins extended to a specified target (1 mm supragingivally) 0.8-1 mm for the shoulder, modified form of the shoulder, and small radius internal angle with a 90° cavosurface margin
Details	20	Adjacent teeth and gingiva are unaffected by the preparation No undercut areas Margins and walls are smooth Margins are continuous and well defined

**Table 2:** The scoring rubric used to assess the intraoral scan stations.

Scoring component	Points	Details
Scanning preparation	25	Order creation is correct The tip is held smoothly and stable No saliva interference during scanning Cleans the lens and waits for 10 s to preheat the lens
Scanning operation	35	Continuous operation of the standard scanning sequence without pauses During the scanning, the lip and other soft tissues are pulled to expand the scanning field Scanning should be completed in 6 min (upper and lower jaws)
Scanning integrity	35	Mesial and distal interproximal surfaces are intact with no missing red-blue data The scan width of the gingival area is at least 2 mm Scanning of the occlusal surface or incisal edge is complete and clear The bite registration is correct
Software tool selection	5	Ability to use the software tools accurately

**Table 3:** The scoring rubric was used to assess the computer-aided design wax pattern stations (crowns).

Scoring component	Points	Details
Order creation	5	Selects preparation in the teeth overview correctly Selects the category correctly (anatomy, wax, and zirconia) Selects the import option correctly Selects the relevant import type correctly Imports the intraoral scan data correctly
Margin	10	Places the margin line correctly Sets the insertion direction correctly
Occlusion	15	Normal overlap and overbite Accurate restoration of the occlusal vertical dimension The occlusion can be checked by dynamic virtual articulation Balanced occlusal forces and no premature contacts
Proximal contact area	10	Correct position and shape of the proximal contact area Correct contact relationship between adjacent teeth
Shape	35	Tooth position: long axis is correctly aligned with the lip and tongue direction, correct proximal and distal orientation, tooth is correctly positioned in the dental arch, and ratio of the tooth length to width is coordinated with that of the adjacent teeth Thickness: the thinnest thickness is not <0.5 mm, and the axial surface thickness is not <1 mm and not >1.5 mm Gingival embrasures are correctly designed and coordinated with those of the adjacent teeth Tooth length: the incisal position is in harmony with that of the adjacent teeth Detailed structure of the surface, such as developmental grooves and ridges Lingual morphology: lingual fossa and marginal ridge morphology
Cement space	5	Acceptable cement space Acceptable extra cement space
Restoration effect	20	Acceptable functionality Acceptable esthetics Acceptable visual harmony

**Figure 4:**
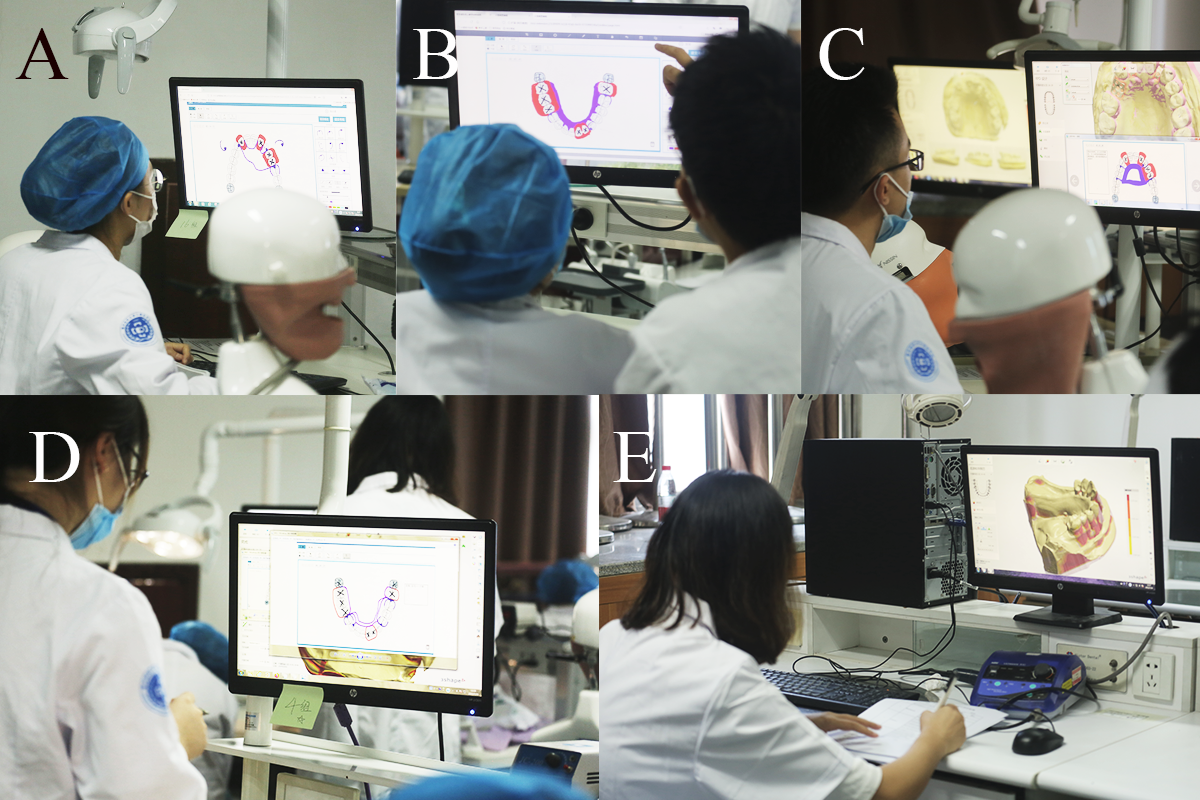
The removable prosthodontics stations of the viOSCE. (A) A dental student designed the framework of a RPD using the Objective Manipulative Skill Examination of Dental Technicians system. (B) Dental and dental technology students discussed the RPD design. (C) A dental technology student created a digital framework of an RPD using the computer-aided design system. (D) A viOSCE examiner scored the first RPD design using the Objective Manipulative Skill Examination of Dental Technician system. (E) A viOSCE examiner scored the final RPD design and the digital framework of the RPD. RPD: removable partial denture; viOSCE: virtual and interprofessional objective structured clinical examination.

At the removable prosthodontics station, real patient cases and intraoral digital models were selected and prepared by the PI, followed by approval by the expert panel. The intraoral digital model was a clinical plaster model scanned using Lab Scanner (E4; 3shape). Each dental student used our previously developed Objective Manipulative Skill Examination of Dental Technicians (OMEDT) system [56] to observe the intraoral digital model and to design a removable partial denture (RPD) framework. At the end of the design task, the dental student submitted the design and then discussed the design with the dental technology student; the dental student could make modifications if they wanted to. Next, each dental technology student used a CAD system (Dental system; 3shape) to design the framework of an RPD on the intraoral digital model based on the final design. A viOSCE examiner scored the first RPD design using the OMEDT system. Next, the viOSCE examiner scored the final RPD design and the digital framework of the RPD. The design discussion station was not scored by a separate examiner (Figure 4 and Tables 4 and 5).

**Table 4:** The scoring rubric used to assess the removable partial denture design stations.

Scoring component	Points	Details
Case observation	20	The missing tooth position is identified accurately and marked correctly on the drawing
Design choices	40	No missing component Indirect retainer is present in the optimal position Design choices do not violate biological principles Clasp choice is optimal for the case The major connector is selected properly with reasonable extension Justified use of clasps and rests
Drawing	20	Ideal drawing Metal components are painted in blue, and resin bases are painted in red
Consistency with task description	10	Exactly as described in the task description Clearly presents the requirements implied in the description, and the design is well aligned with the corresponding description Gives consideration to both esthetics and functions
Neatness and accuracy in presentation	10	Neat and accurate No inconsistencies between the table and drawing

**Table 5:** The scoring rubric used to assess the computer-aided design wax pattern stations (removable partial denture [RPD] framework).

Scoring component	Points	Details
Order creation	5	Selects artificial teeth in the teeth overview correctly Selects the category correctly (removable—RPD frame) Selects the import option correctly Selects the relevant import type correctly Imports the laboratory scan data correctly
Surveying	10	Insertion direction is correctly chosen Undercuts are correctly identified
Virtual cast preparation	20	Correct paralleled blockout Correct shaped blockout Correct arbitrary blockout Correct relief setting
Framework design	40	Reasonable position and shape of clasp Reasonable position and shape of rest Reasonable position and shape of major connector Reasonable position and shape of retention grid Reasonable position and shape of finishing line
Form	25	All parts are connected as a whole The thickness and strength of the framework meet the requirements The thickness is uniform, and the surface is smooth Esthetics are acceptable

The clinical diagnostics station used a virtual standardized patient (VSP) with the haptic device (UniDental, Unidraw). The VSP hardware does not have an anthropomorphic shape, but it interacts through vocal, visual, and haptic devices. On the basis of the novel oral knowledge graph and the coupled, pretrained Bert models, the VSP can accurately interact with a dentist’s underlying intention and express the symptom characteristics in a natural style [57]. On the basis of this algorithm, the PI adjusted and entered the real patient case details, allowing the dental technology student to work with the dental student as a chairside dental technician to make a diagnosis based on the information obtained from the interactions with the VSP. In this study, the clinical case designed on the VSP was a patient who required root canal treatment and full crown restoration. At the end of the dental student’s diagnosis and simulation, the dental technology student was required to assist the dental student in designing the restoration plan and help the patient in choosing the materials for crown restoration (this often determines the price of the treatment). Thus, dental and dental technology students finalized the prosthodontic treatment plan collaboratively. The visual device built a virtual dental clinic environment and VSP model, allowing the students to view the VSP from global, extraoral, and intraoral perspectives. The haptic device allows dental students to perform intraoral and extraoral examinations using essential tools to explore the diagnostic evidence.

**Figure 5:**
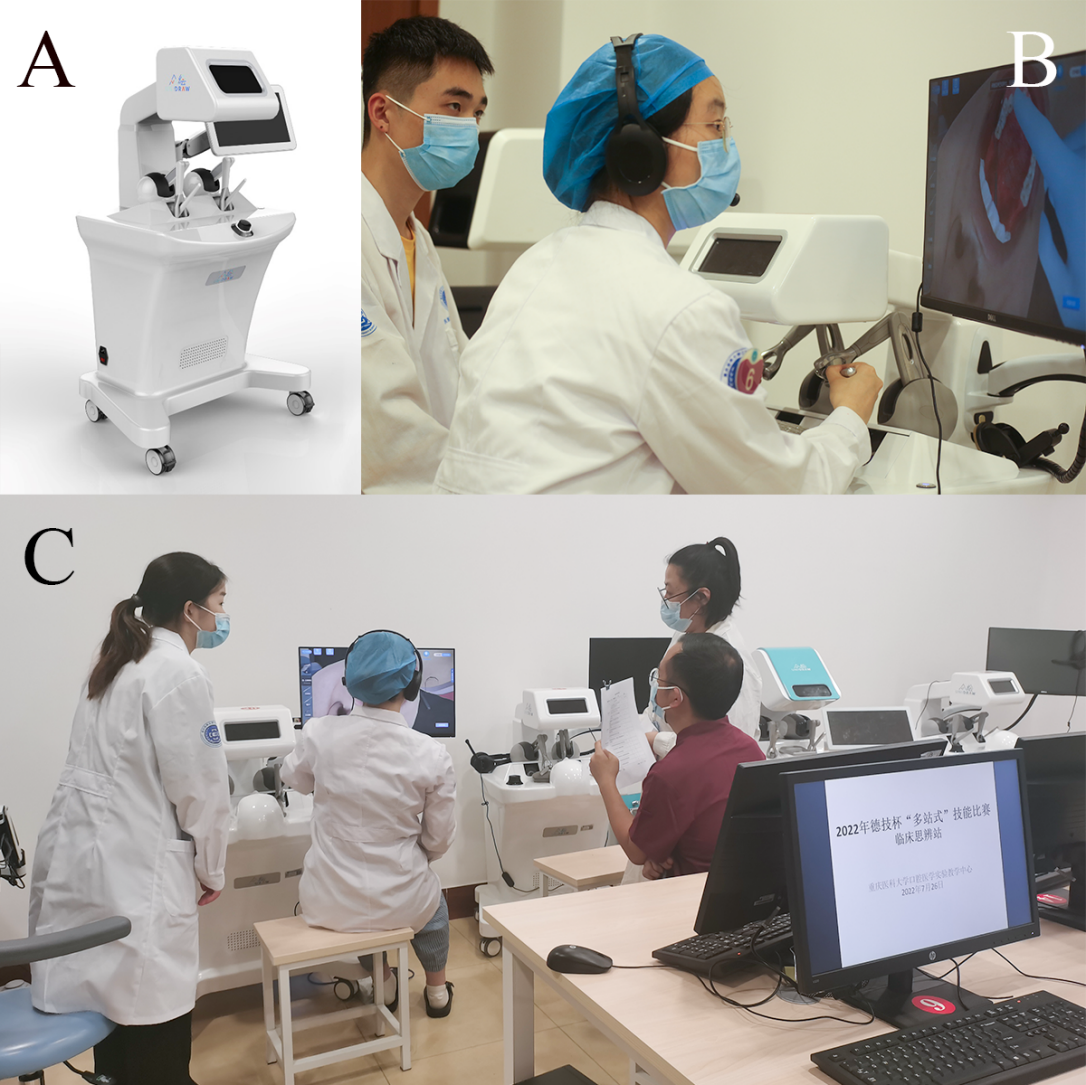
The clinical diagnostics station of the viOSCE. (A) The VSP with the haptic device, UniDental. (B) Dental and dental technology students performed intraoral palpation on the VSP using the haptic device. (C) Then, 2 viOSCE examiners scored the process and clinical diagnostic results. viOSCE: virtual and interprofessional objective structured clinical examination; VSP: virtual standardized patient.

Owing to the complexity of collaborative diagnosis, the station was manually scored by 2 examiners independently based on the previously developed scoring rubrics, whereas the UniDental output machine provided an additional score according to the previously developed scoring rubrics. The average of the 3 scores formed the final score for the station. To ensure the relative independence and internal consistency of all scores, the examiners were not informed about the existence of the machine score. The PI exported the machine score data from the VSP at the end of the experiment (Figure 5 and Table 6).

**Table 6:** The scoring rubric used to assess the clinical diagnostic stations.

Scoring component	Points	Details
History taking	25	The content of the inquiry is accurate Few questions unrelated to the disease or clinical situation Inquiries are made sequentially, purposefully, and hierarchically
Intraoral examination	25	Tool selection is accurate Humanistic care is reflected during the examination Appropriate oral examination items are performed based on the case information
Auxiliary examination	10	Correct auxiliary examination items are selected Correct interpretation of auxiliary examination results
Case analysis	20	Correct diagnosis of the case Correct selection of the diagnostic criteria Correct differential diagnosis of the case Correct selection of the basis for differential diagnosis
Plan design	20	Correct treatment plan design according to the disease condition Provides advice about material selection based on patient request

### Performance Evaluation of viOSCE

In this study, fourth-year undergraduate dental students and third-year undergraduate dental technology students participated in viOSCE because students at this stage of education had completed preclinical professional training. Overall, 50 students who met these requirements, including 25 (50%) dental students and 25 (50%) dental technology students, were recruited into the viOSCE user evaluation experiment and were divided into groups of 2 comprising 1 dental student and 1 dental technology student. The PI and examiner teams did not influence or determine the team-formation process. All participating students were informed that as this viOSCE was in the experimental phase, it was conducted as a small extracurricular skills competition, thus allowing for self-evaluation without a final examination situation, as previously reported [58]. This approach allowed for the simulation of an examination situation without affecting the final examination grade of the students. A month before commencing the experiment, the PI led an web meeting for students to explain the viOSCE, the relevant knowledge points, and the need to practice fully during the upcoming month. At the end of the meeting, the students completed the Readiness for Interprofessional Learning Scale (RIPLS) pretest questionnaire, which is a 19-item 5-point Likert-scale questionnaire; this type of questionnaire is the most frequently used method for the subjective evaluation of IPE and IPCP [18].

viOSCE was piloted after the 1-month preparation period. The panel of examiners marked points according to the previously prepared scoring rubrics, whereas some of the points were automatically scored by a machine. After this step, the participating students completed the posttest self-made questionnaire, to which a mutual evaluation scale and a viOSCE evaluation scale were added. The mutual evaluation scale asked the students to score the performance of their partner, whereas the viOSCE evaluation scale asked the students to score viOSCE. In total, 6 items were included in the mutual evaluation scale, and 7 items were included in the viOSCE evaluation scale (Textboxes 1 and 2). All items in both questionnaires were set to a maximum score of 100. Before issuing the questionnaire, the panel first reviewed all the questions, clarified ambiguities, and removed any double-barreled questions [59,60]. At the end of the experiment, one-on-one interviews were conducted with all the students to determine their perceptions about viOSCE.

**The mutual evaluation scale administered to dental and dental technology student groups who participated in the virtual and interprofessional objective structured clinical examination (viOSCE).**

**Items**

- Final contribution
- Person-organization fit
- Performance in viOSCE
- Professional skill
- Practice volume before viOSCE
- Motivation to participate

**The virtual and interprofessional objective structured clinical examination (viOSCE) evaluation scale administered to dental and dental technology student groups who participated in viOSCE.**

**Items**

- Evaluation of viOSCE effectiveness
- Evaluation of equipment, network operation and maintenance
- Evaluation of viOSCE examiners
- Evaluation of viOSCE staff
- Rationality of the clinical diagnostic design
- Rationality of the fixed prosthodontics design
- Rationality of the removable prosthodontics design

### Statistical Analysis

Data were tabulated in a Microsoft Excel spreadsheet and imported into IBM SPSS Statistics for Windows (version 26.0; IBM Corp) for descriptive analysis. GraphPad PRISM 8.0 software (GraphPad Software) was used to create the graphs. Responses were summarized, and comparisons were made. Output data were presented as percentages and in graphical format. The Shapiro-Wilk test was used to test for normal distribution. Specific data analysis tests performed included descriptive statistics, 2-tailed paired *t* tests, and correlation analyses.

### Ethical Considerations

The research ethics committee of the Affiliated Hospital of Stomatology, Chongqing Medical University, approved this study protocol (COHS-REC-2022; LS number: 096). All participants provided written informed consent before participation in the study.

## Results

Of the 50 students, 32 (64%) completed the experiment. Interviews were conducted with the students who dropped out of further participation in the study. The main reasons for dropping out included the students’ belief that they or their collaborating partners had not practiced sufficiently to perform well in the experiment. A group had a verbal confrontation approximately an hour before the experiment began. The main reason for the conflict was that the dental technology student accused the dental student of not practicing sufficiently before the experiment. According to the study protocol, at the end of the experiment, the conflict was resolved by the PI. Both parties were counseled, mediated by the PI, and the 2 parties reconciled.

**Figure 6:**
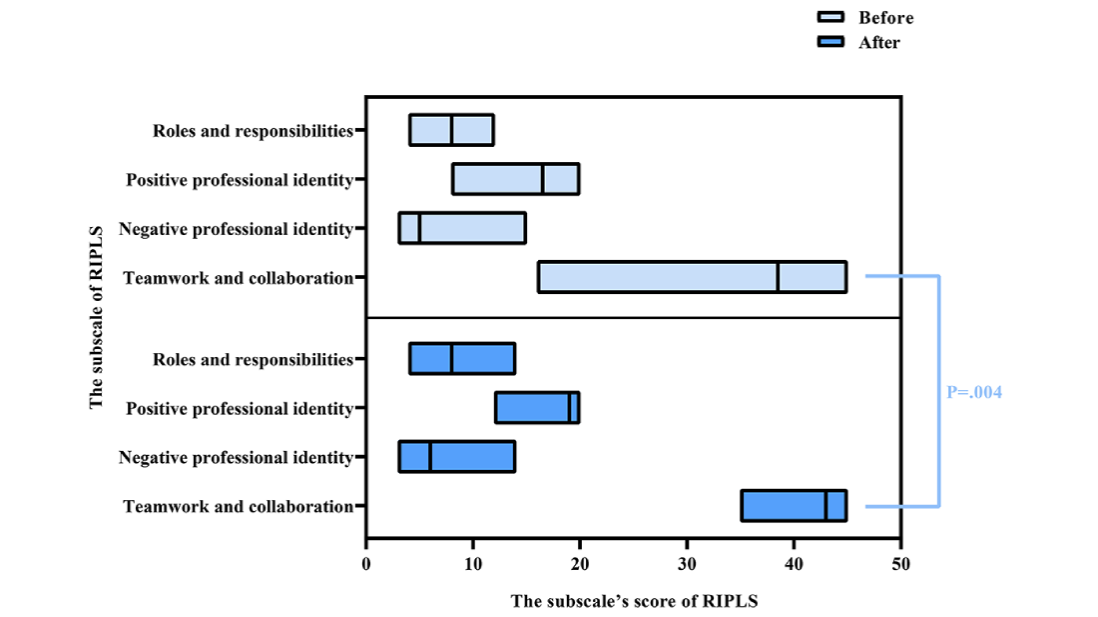
RIPLS data before and after the virtual and interprofessional objective structured clinical examination pilot study. RIPLS: Readiness for Interprofessional Learning Scale.

Data from the RIPLS, mutual evaluation scale, and viOSCE evaluation scale were first analyzed to determine the impact of viOSCE on the subjective evaluation of IPCP. All students (32/32, 100%) who completed the experiment were administered the RIPLS questionnaire before and after the experiment. The Cronbach α values were .835 for the pretest data and .731 for the posttest data, suggesting that the reliability and internal consistency were acceptable. The results failed the Shapiro-Wilk test for normality; therefore, the data were analyzed using the Wilcoxon signed rank test. The teamwork and collaboration subscale scores were significantly increased after the experiment (*P*=.004). In addition, there was an nonsignificant decrease in the negative professional identity subscale scores (*P*=.21). There was also an insignificant increase in the scores on the positive identity subscale and on the roles and responsibilities subscale (*P*=.13 and *P*=.96, respectively). Figure 6 depicts the RIPLS data before and after the viOSCE pilot.

**Figure 7:**
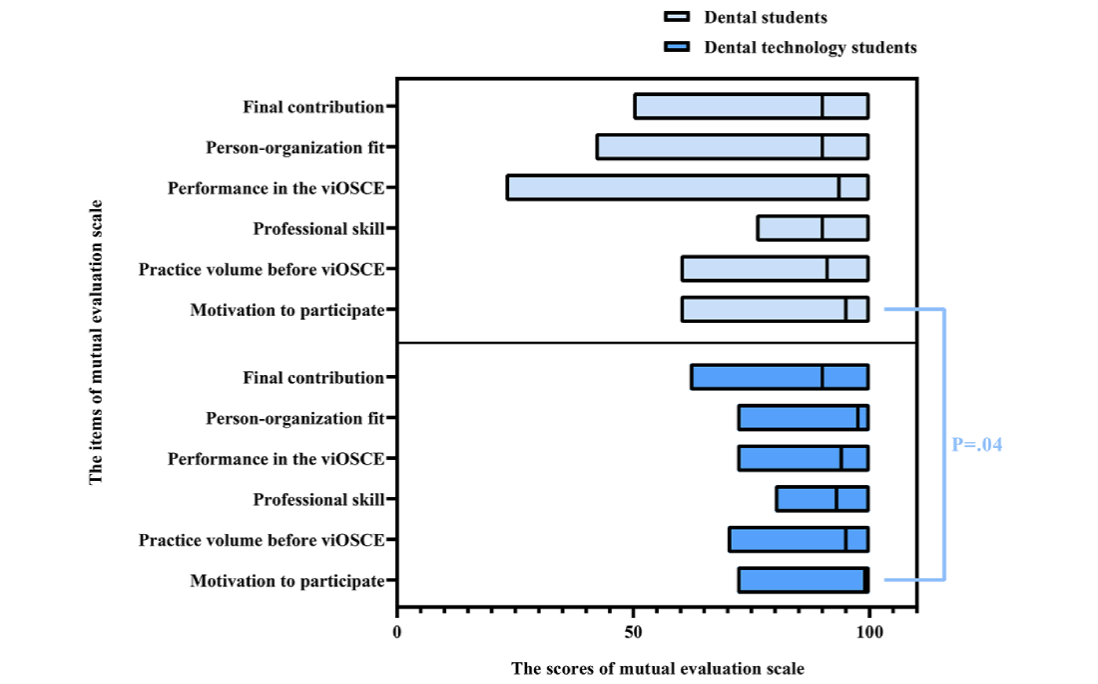
Mutual evaluation scale data for the dental and dental technology students. viOSCE: virtual and interprofessional objective structured clinical examination.

After the experiment, the mutual evaluation scale was administered to all participating students (32/32, 100%) who completed the experiment. The Cronbach α value was .873, suggesting good reliability and internal consistency. Comparison of the results of the dental and dental technology students revealed that only the mutual evaluation scores for competition motivation were significantly different between the 2 groups (*P*=.04). The dentistry and dental technology students evaluated each other’s motivation to participate in the competition (competition motivation), and the dental students had higher scores than the dental technology students. Figure 7 depicts the mutual evaluation scale scores of the dental and dental technology students.

**Figure 8:**
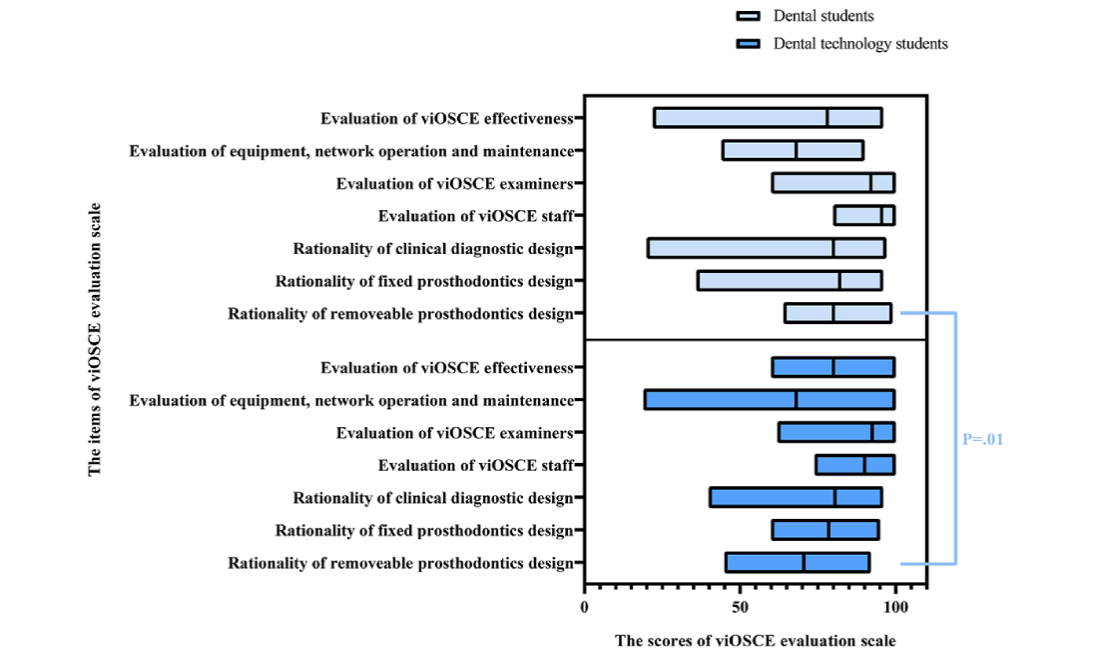
viOSCE evaluation scale data for dental and dental technology students. viOSCE: virtual and interprofessional objective structured clinical examination.

Similarly, after the experiment, the viOSCE evaluation scale was administered to all students (32/32, 100%). The Cronbach α value was .706, suggesting acceptable reliability and internal consistency. Comparison of the viOSCE evaluation scale results of the dental and dental technology students with the Wilcoxon signed rank test results revealed that only the evaluation scores for the removable prosthodontics design were statistically significant (*P*=.01) among the 7 items. Figure 8 depicts the viOSCE evaluation scale scores of the dental and dental technology students.

**Figure 9:**
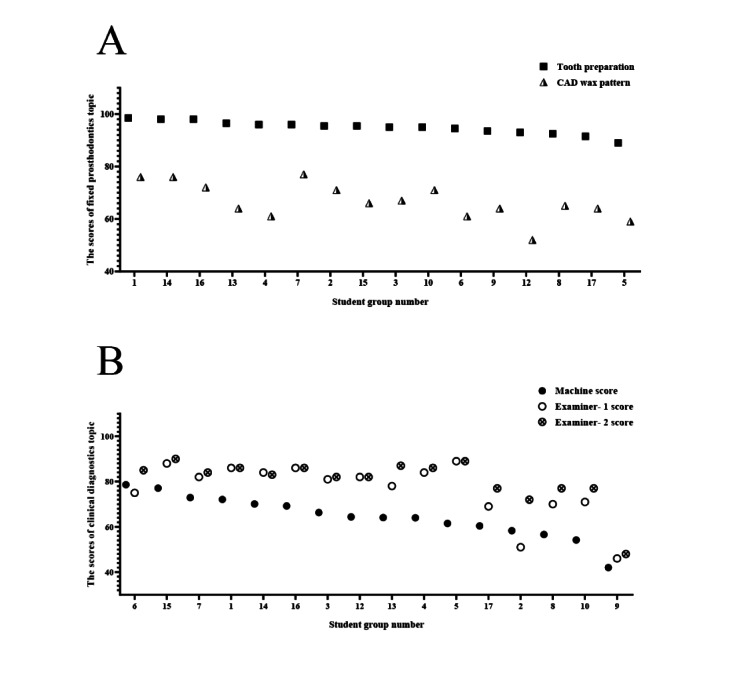
Spearman correlation analysis of the virtual and interprofessional objective structured clinical examination scores. (A) For the fixed prosthodontics topic, the positive correlations between the scores of the tooth preparation station and the CAD wax pattern station were significant. (B) For the clinical diagnostics topic, the positive correlations between the virtual standardized patient machine score and the examiners’ scores were significant. CAD: computer-aided design.

To explore the validity of the examiner panel scores in viOSCE, correlation analysis was conducted on the scores of each station under the 3 topics. Using Spearman correlation coefficient, for the fixed prosthodontics topic, a strong positive correlation between the scores of the tooth preparation station and the CAD wax pattern station was noted, and it was statistically significant (*r*=0.67; *P*=.005). Positive correlations between the scores of the intraoral scan station and the CAD wax pattern station and between the intraoral scan station and the tooth preparation station were not statistically significant (*r*=0.179; *P*=.51 and *r*=0.387; *P*=.14, respectively). For the removable prosthodontics topic, 11 (69%) of the 16 student groups finally decided to modify the RPD design initially made by the dental students. A negative but statistically insignificant correlation between the scores of the RPD design station and the CAD wax pattern station was noted (*r*=−0.111; *P*=.68). For the clinical diagnostics topic, the correlation analysis was conducted primarily for the machine scores and the examiner scores to determine the usability of the VSP in viOSCE and the consistency of machine scoring and examiner scoring. The results revealed a significant positive correlation between the scores of the 2 examiners, and the positive correlation between the machine scores and the 2 examiners’ scores was also significant. The results are shown in Table 7 and Figure 9.

**Table 7:** Spearman correlation analysis of the virtual and interprofessional objective structured clinical examination scores.

Topic and station		Correlation coefficient	*P* value (2-tailed)	Participants (n=16), n (%)
**Fixed prosthodontics**				
	Tooth preparation vs CAD^a^ wax pattern	0.670	.005^b^	16 (100)
	Intraoral scan vs tooth preparation	0.387	.14	16 (100)
	Intraoral scan vs CAD wax pattern	0.179	.51	16 (100)
**Removable prosthodontics**				
	RPD^b^ design vs CAD wax pattern	−0.111	.68	16 (100)
**Clinical diagnostics**				
	Machine score vs examiner-1 score	0.601	.01^d^	16 (100)
	Machine score vs examiner-2 score	0.629	.009^b^	16 (100)
	Examiner 1 score vs examiner-2 score	0.855	<.001^e^	16 (100)

^a^CAD: computer-aided design.

^b^RPD: removable partial denture.

**Figure 10:**
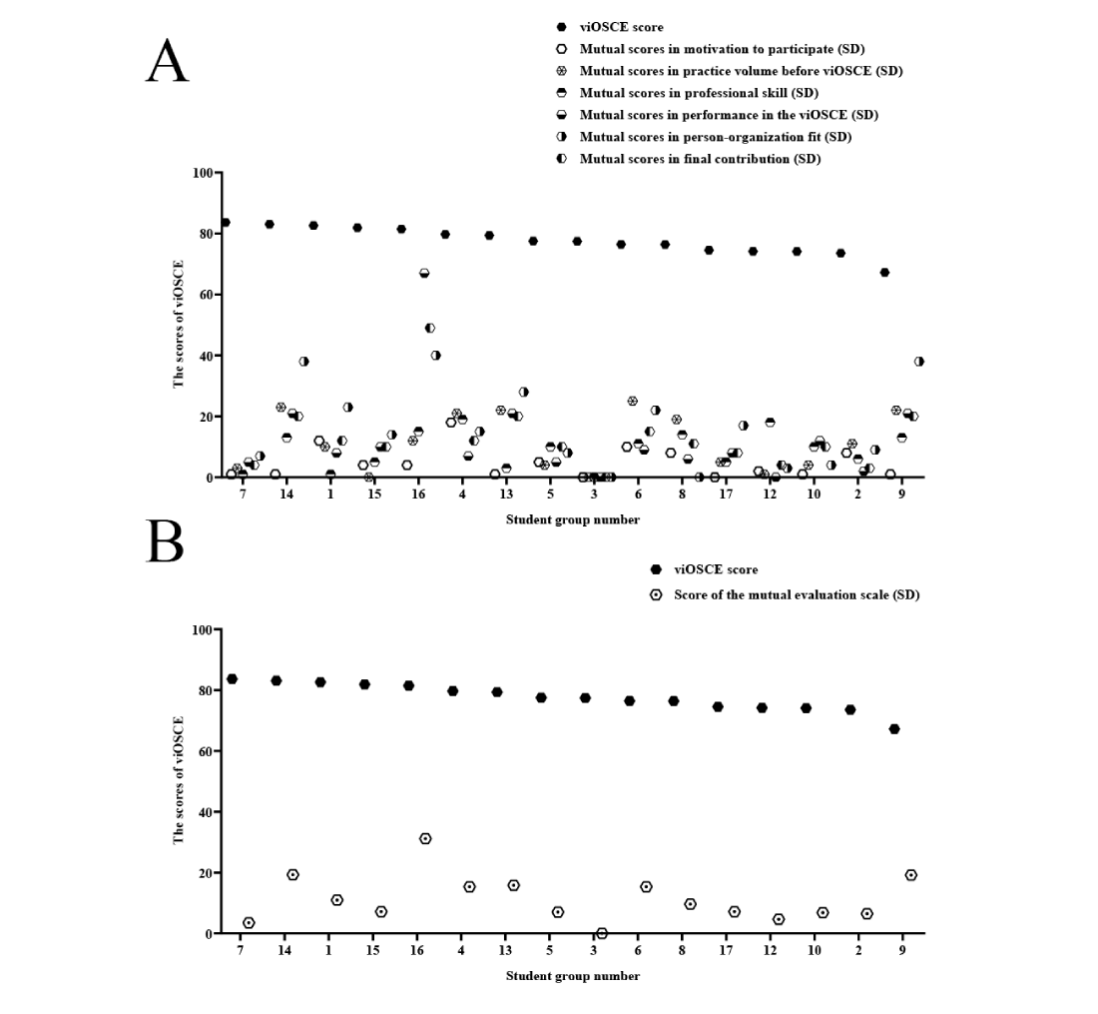
Correlation analysis of the viOSCE scores and the mutual evaluation scale. (A) Correlation analysis of the viOSCE scores and the SD of the scores for each item on the mutual evaluation scale. (B) Correlation analysis of the viOSCE scores and the SD of the mean scores on the mutual evaluation scale. viOSCE: virtual and interprofessional objective structured clinical examination.

To explore the relationship between the objective and subjective evaluations, correlation analysis was conducted between the viOSCE scores and the RIPLS scores as well as between the viOSCE scores and the mutual evaluation scale scores. Insignificant negative correlations were noted between the subjective evaluation scores presented by RIPLS and viOSCE. Similarly, the correlation of the mutual evaluation scale score with the viOSCE scores was not significant. The SD of the scores on the mutual evaluation scale showed a decreasing trend among students with higher viOSCE scores and those with lower scores, but an increasing trend was observed among those with median scores (Table 8 and Figure 10).

**Table 8:** Spearman correlation analysis between the subjective and objective evaluations presented by the Readiness for Interprofessional Learning Scale (RIPLS) and mutual evaluation scale.

	Correlation coefficient	*P* value (2-tailed)	Participants (n=16), n (%)
The examiner panel scores of viOSCE^a^ vs the intragroup mean score of RIPLS (before the test)	−0.272	.15	16 (100)
The examiner panel scores of viOSCE vs the intragroup mean score of RIPLS (after the test)	−0.302	.13	16 (100)
The examiner panel scores of viOSCE vs the intragroup mean score of the mutual evaluation scale	−0.038	.44	16 (100)

^a^viOSCE: virtual and interprofessional objective structured clinical examination.

In the one-on-one interviews, 29 (91%) of the 32 students approved of the effectiveness of viOSCE and wanted to use it to assess their IPCP ability in the graduation examination. At the fixed prosthodontics station, 56% (9/16) of the dental technology students complained about the lack of lingual space prepared by their partners at the tooth preparation station, which made it difficult to design crown wax patterns, and the corresponding dental students reported not being aware of the condition before viOSCE. At the removable prosthodontics station, almost all the dental students (15/16, 94%) reported that the advice given by the dental technology students was effective in helping them complete the RPD design and considered their design practice to be insufficient. In contrast, the dental technology students reported that helping the dental students complete the RPD design made them feel satisfied with their professional competence and felt that they were truly part of the team during the collaboration. At the clinical diagnosis station, the dental students felt that their clinical practice experience was not sufficient, especially when the dental technology students could provide a diagnostic plan faster than themselves.

In terms of positive feedback, the students believed that viOSCE promoted the friendship between themselves and their partners, helped them realize the continuity and relevance between their own work and the work of their partners, and enabled them to acquire a deep understanding of IPCP. The negative feedback mainly focused on their lack of clinical knowledge, inadequate preparation, and long waiting time at some stations.

## Discussion

### Principal Findings

The IPCP results of dentists and dental technicians reflect the quality of their IPE, skill training, and clinical experience. The results contribute to the much-needed IPE assessment literature and suggest that teamwork skills can be improved by IPCP and effectively assessed using this new evaluation scale. We used a modified Delphi process in this study. This is in accordance with Simmons et al [27], who found that the modified Delphi process is an effective tool to obtain consensus among professionals for the foundational work required. In addition, our study demonstrated the effectiveness of iOSCE in asynchronous and synchronous collaboration scenarios, while providing a methodological reference for developing a new iOSCE for dental health care professionals. As the collaboration scenario between the dentist and the dental technician may be both asynchronously applied through prescriptions and synchronously conducted in chairside discussions [61-63], it was deemed appropriate for the viOSCE framework to consider both synchronous and asynchronous scenarios.

The viOSCE scores in this study also reflect the effectiveness of the framework design. From the viOSCE examiner scores in the fixed prosthodontics section, a significant positive correlation between the scores of the tooth preparation station and the CAD wax pattern station was evident. This finding is consistent with the actual clinical asynchronous delivery scenario, where the dentist’s preparation largely determines the quality of the dental technician’s crown wax pattern. Qualitative evaluations extracted from the one-on-one interviews also supported this result. For the removable prosthodontics section, the negative correlation between the scores of the RPD design station and the CAD wax pattern station was not statistically significant, which might be owing to the fact that more than half of the groups (15/16, 94%) worked collaboratively to modify the RPD design to possibly compensate for the lack of training, which is consistent with the findings about dentists’ inadequate competence in RPD design reported in other studies [64,65].

As OSCE is essentially a simulated scenario-based examination, the use of virtual technology to build simulated scenarios has become an important direction for OSCE-related studies, especially in the field of dental education [43]. The COVID-19 pandemic has further contributed to dental educators’ interest in this area, as dental clinical practice typically occurs in a virus-laden aerosolized environment [66]. Therefore, providing a safe and robust learning environment in the simulation clinic is also critical to help students compensate for lost educational time. The virtual technologies used to construct the simulated clinical environment in this study include VSP and CAD. Previously, Janda et al [67] developed a virtual patient as a supplement to standard instruction in the diagnosis and treatment planning of periodontal disease. However, it could not fully understand complex or ambiguous questions, and the students felt frustrated during the practice [67].

Tanzawa et al [68] developed a robot patient that could reproduce an authentic clinical situation and introduced it into OSCE. However, the dialogue recognition of the robot patients was prespecified; the robot was unable to identify subjective patient descriptions or the dentist’s interrogation intention and could not support intraoral or extraoral examinations to obtain diagnostic evidence [68]. To fill these gaps, our study used VSP with intention recognition and haptic feedback to construct virtual dental clinical practice and diagnosis scenarios more realistically. As the diagnostic evidence collected by students through interrogation, inspection, and palpation was automatically summarized for the final differential diagnosis, and omissions in the examination process eventually led to a misdiagnosis, the system simulated a high-fidelity clinical environment. In addition, the results showed that 1 (6%) of the 16 student groups misdiagnosed their VSP because of incomplete interrogation and palpation. The correlations between the scores of the 2 examiners and the machine scores were statistically significant, thus confirming the robustness of the high-fidelity simulation scenarios constructed by the VSP and the machine scores. On the basis of these results, the use of VSP should be expanded and integrated into daily teaching to give students more opportunities for clinical practice training.

Consistent with the results of previous OMEDT studies [56], the use of CAD technology in viOSCE significantly reduced the time spent at each station for the dental technology students. Some dental technology students complained about the slowness of the CAD program. Upon further investigation, it was found that they imported both impressions at the same time. In dental laboratory practice, dental technicians usually import the impressions separately to prevent computational issues. This finding exposes the lack of virtual dental laboratory practice skills in teaching, which needs to be addressed.

The results showed that the teamwork and collaboration subscale scores were significantly increased at the end of the study (*P*=.004), suggesting that viOSCE can improve students’ teamwork skills. The increase in the other 3 subscale scores, although not statistically significant, can be explained by the choice of timing of viOSCE. The optimal time to expose medical students to IPE is still subject to debate [18]. viOSCE, as a clinical IPCP intervention introduced during the clinical year, had no significant effect on the promotion of negative or positive identity or roles and responsibilities. This finding may be due to the fact that the students’ professional cognition had been stereotyped at this time, making it difficult to effect significant changes through IPE or IPCP intervention. This conclusion is supported by a previous study [69].

The results of the mutual evaluation scale showed statistically significant difference in participant motivation between the 2 professional groups, which could be explained by the results of the roles and responsibilities subscale. Of the 16 dental technology students, 4 (25%) expressed that they would not practice as dental technicians in the future because they wanted to choose other careers. The differences in the scores of the other items were not statistically significant, thus showing the effectiveness of viOSCE in the development of teamwork spirit. This result confirms that the OSCE design is well suited as a final evaluation of IPE and IPCP. In addition, the average score of each item of viOSCE was >60, indicating that the students were satisfied with the design and operation of viOSCE. The differences in scores between the 2 types of professionals were not statistically significant, except at the removable prosthodontics station, which was probably caused by the dental technology students’ unfamiliarity with the CAD program.

Overall, the internal consistency of all subjective evaluations was acceptable, and the results met expectations. Interesting observations were also made regarding the correlation between the subjective and objective evaluations. The SD of the scores on the mutual evaluation scale showed a decreasing trend among the dental and dental technology students with higher viOSCE scores and those with lower scores, but an increasing trend in the median score was observed. Although this trend was not statistically significant due to sample size limitations, this early finding provides data support for a summary of clinical experience published previously by Preston [70], who reported that the intensity of the relationship between dentists and dental technicians is determined by the difference in their professional skills. If the professional skills of both parties are high, there will be few problems in their cooperative relationship. The more discriminating and demanding the technician or dentist becomes, the more the relationship is strained when either fails to perform up to the other’s standards. This result suggests that in the study of IPE and IPCP for dentists and dental technicians, it is not sufficient to explore the improvement of the traditional assessment dimensions such as team collaboration skills and identity. The final quality of the output must be included in the assessment dimension. This also reaffirms the effectiveness of viOSCE as an objective, quantitative evaluation tool for IPE and IPCP.

### Limitations and Future Studies

The main limitation of our study is the small convenience sample of participating students, which could have led to self-selection bias. The sample size should be expanded in the future to obtain more data and to further verify the robustness of the viOSCE framework. In addition, whether viOSCE should be made a part of the large and more complete OSCE to test the ability of students to meet undergraduate graduation requirements will also be the focus of our next study. Moreover, the independent application of the novel VSP in the education of dental students is an interesting topic that will be explored in the next step of this study.

### Recommendations

On the basis of our results, we provide the following recommendations:

1. All dental health professionals should be educated to deliver patient-centered care as members of an interdisciplinary team [16].
2. IPE intervention–related skills should be introduced as preclinical skills.
3. The cooperation of the dental care team is complex, and the training for improving the cooperation ability of the dental care team should include both subjective and objective assessments.
4. viOSCE and scale assessment should be introduced for the assessment of IPE and IPCP at the clinical stage of training.

### Conclusions

In this study, a novel viOSCE framework was developed and piloted. Data based on subjective evaluation scales and objective examiner scores were collected and analyzed, confirming the effectiveness of viOSCE as an objective evaluation tool for IPE and IPCP. The experimental design should be expanded to include more randomly selected students with a scientifically determined sample size to further develop studies focused on IPE and IPCP in dentistry and dental technology, ultimately promoting quality in dental clinical practice.

## Abbreviations

CAD: computer-aided design
IPCP: interprofessional collaborative practice
IPE: interprofessional education
OMEDT: Objective Manipulative Skill Examination of Dental Technicians
OSCE: objective structured clinical examination
PI: principal investigator
RIPLS: Readiness for Interprofessional Learning Scale
RPD: removable partial denture
viOSCE: virtual and interprofessional objective structured clinical examination
vOSCE: virtual objective structured clinical examination
VSP: virtual standardized patient

## Data Availability

The data sets generated and analyzed during this study are available from the corresponding author upon reasonable request.
